# The Impact of Health Policies and Sociodemographic Factors on Doubling Time of the COVID-19 Pandemic in Mexico

**DOI:** 10.3390/ijerph18052354

**Published:** 2021-02-28

**Authors:** Lina Díaz-Castro, Héctor Cabello-Rangel, Kurt Hoffman

**Affiliations:** 1 Direction of Epidemiological and Psychosocial Research, National Institute of Psychiatry Ramón de la Fuente Muñiz, Mexico City 14370, Mexico; 2 Diagnostic Auxiliary Division, Psychiatric Hospital Fray Bernardino Álvarez, Mexico City 14000, Mexico; hector19.05.19.05@gmail.com; 3 Research Center in Animal Reproduction, Autonomous University of Tlaxcala, Tlaxcala 90000, Mexico; rexvitro@hotmail.com

**Keywords:** health policy, SARS-CoV-2, doubling time

## Abstract

*Background*. The doubling time is the best indicator of the course of the current COVID-19 pandemic. The aim of the present investigation was to determine the impact of policies and several sociodemographic factors on the COVID-19 doubling time in Mexico. *Methods*. A retrospective longitudinal study was carried out across March–August, 2020. Policies issued by each of the 32 Mexican states during each week of this period were classified according to the University of Oxford Coronavirus Government Response Tracker (OxCGRT), and the doubling time of COVID-19 cases was calculated. Additionally, variables such as population size and density, poverty and mobility were included. A panel data model was applied to measure the effect of these variables on doubling time. *Results*. States with larger population sizes issued a larger number of policies. Delay in the issuance of policies was associated with accelerated propagation. The policy index (coefficient 0.60, *p* < 0.01) and the income per capita (coefficient 3.36, *p* < 0.01) had a positive effect on doubling time; by contrast, the population density (coefficient −0.012, *p* < 0.05), the mobility in parks (coefficient −1.10, *p* < 0.01) and the residential mobility (coefficient −4.14, *p* < 0.01) had a negative effect. *Conclusions*. Health policies had an effect on slowing the pandemic’s propagation, but population density and mobility played a fundamental role. Therefore, it is necessary to implement policies that consider these variables.

## 1. Introduction

On 30 January 2020, the World Health Organization (WHO) declared a Public Health Emergency of International Concern (PHEIC) for the illness COVID-19, caused by a new coronavirus designated Severe Acute Respiratory Syndrome Coronavirus-2 (SARS-CoV-2) [1]. On 28 February 2020, the Secretary of Health (SSA, acronym in Spanish; Secretaría de Salud), the governing body of the National Health System (SNS, acronym in Spanish; Sistema Nacional de Salud), reported the first three cases of COVID-19 in Mexico [2]. Up to the day of this writing, 30 November 2020, Mexico has registered a total of 1,107,071 cases of COVID-19, resulting in 105,655 deaths [3]. 

In accordance with the Strategic Preparation and Response Plan for COVID-19 of the WHO [4], the SSA implemented several policies for confronting and mitigating the pandemic’s impact [2]. In the present context, policies are understood as actions taken by the government with the objective of advancing the public’s interests, arising from decisions grounded on diagnostic processes and feasibility analysis, in order to attend effectively to specific public problems, such as the current COVID-19 pandemic [5].

The policies that have been implemented can be generally classified as those involving closures or containment, economic policies that include monetary stimuli and debt assistance and those targeting the health system, such as investments in health and public information campaigns, among others [6]. In Mexico, the principal objective of these policies has been to flatten the contagion curve, slowing the propagation velocity of SARS-CoV-2, thus avoiding the saturation of cases in hospitals.

In the literature, it has been reported that policies aimed at certain sociodemographic conditions such as poverty and income level had only a minimal mitigating effect on the pandemic [7,8]. On the other hand, sociodemographic characteristics also impact on the mobility of the population, and greater mobility has been associated with a greater velocity of viral propagation [9].

The velocity of virus propagation is operationally defined by the “doubling time”: the number of days required for the number of cases of an infection, such as the current pandemic of COVID-19, to double in size, taking as a base indicator the increase in the accumulated number of cases. The doubling time is the best epidemiological tracer of the course of a pandemic [10], measuring the rate at which viral transmission is increasing or decreasing. Determining the transmission velocity of SARS-CoV-2 is fundamental for evaluating the impact of intervention measures and implemented policies. In regard to the SARS-CoV-2 pandemic, studies have reported that the doubling time ranged from 7.4 days in the initial phases of the pandemic [11] to 2.5 in the continuous phases of propagation [12]. In a recent study [13] that reported the doubling times of the current pandemic in various countries, it was mentioned that the doubling time in Mexico was low, but at 135 days, the COVID-19 cases and deaths began to accumulate more rapidly. However, we found no published studies on the relationship between doubling time and the implementation of policies and how this might vary among the 32 Mexican states. 

The objective of the present study was to measure the impact of policies that have been implemented in response to the COVID-19 health emergency on the velocity of viral transmission as reflected by the doubling time, considering the mobility and sociodemographic characteristics in each of the 32 Mexican states.

## 2. Materials and Methods

### 2.1. Study Design

A retrospective longitudinal study was carried out across the months of March to August of 2020. We used data from the National System of Epidemiological Vigilance (SINAVE, acronym in Spanish; Sistema Nacional de Vigilancia Epidemiológica) for COVID-19 in Mexico, published within the official website of the SSA, which provides daily reports of the number of cases at the national level as well as for each federal entity [2]. We used weekly measures of new cases and accumulated cases in order to correspond them to the weekly publications of policies in the Official Journal of the Federation (DOF, Diario Oficial de la Federación). The outcome variable was the number of days required for the number of cumulative COVID-19 cases to double in size (doubling time) [11]. The principal independent variable was the policy index (see below), derived from the number and type of policies implemented during each epidemiological week and published online by the DOF. Considering the demographic and socioeconomic context of each of the Mexican states, other variables that could have influenced the doubling time were considered in the models, such as population size and density, health index, poverty, income per capita and mobility variables.

### 2.2. Study Variables

#### 2.2.1. Outcome Variable: Doubling Time

Equation (1) was used for estimating the doubling time at the end of each epidemiological week:

(1) $$Dt_{i}=\left(w_{i}-w_{i-1}\right)\frac{ln\left(2\right)}{ln\left(\frac{c_{i}}{c_{i-1}}\right)}$$

where $w_{i}-w_{i-1}$ is the difference in days between epidemiological weeks (7 days);

$c_{i}$ is the number of accumulated cases during the week i;

$c_{i-1}$ is the number of accumulated cases in the previous epidemiological week. 

Since our study analyzed the cumulative case numbers from epidemiological weeks 13 to 36, we have estimates of doubling time beginning on epidemiological week 13 + 1. 

#### 2.2.2. Independent Variables

Policy Index. Data on public health policies issued in response to the COVID-19 pandemic were compiled from publicly available official sources, such as government web pages and publications of the DOF of each of the Mexican states (Supplementary File S1). A complete survey of all relevant published documents was undertaken, and each of the COVID-19 response policies was classified into one of the three categories: (1) closures and containment, (2) economic measures and (3) health measures (see Table 1). We then selected those policies that were in effect during the epidemiological weeks in question (weeks 13 to 36).

The policy index takes into consideration the number of policies implemented during each epidemiological week, adjusted for their relative values. In order to assign relative values to each of the policies according to their potential relevance for mitigating viral transmission, each policy was scored on a scale of 0 to 10 (0 = not relevant) by a panel of 10 multidisciplinary experts in public health (Supplementary File S2). The mean score for each policy was calculated, and only those policies with an average score greater than 7 (a total of 10 policies; see Table 1) were considered in the calculation of the policy index. We then used a common way to create a composite “policy index”—a simple multiplicative index, according to the Oxford COVID-19 Government Response Tracker (OxCGRT), which offers a systematic manner for carrying out a rigorous tracking of governmental COVID-19 responses in all countries and times [14]. 

Population Size and Density. The population size and density for each state were derived from the database of the National Population Council (CONAPO, acronym in Spanish; Consejo Nacional de Population) [15]. 

Health Index. The “health index” was calculated for each state as recommended by the United Nations Development Programme (UNDP) [16], considering the state’s mean life expectancy, along with maximum (85 years) and minimum (25 years) life expectancy values defined by the UNDP. Thus:

Health index of the state = (mean life expectancy for the state − 25)/(85 − 25)

(2)

Poverty. Estimations of poverty in each Mexican state considered the percent of the population with income below the poverty line, which implies the presence of at least one indicator of a lack of social necessities (education, health services, social security, food, a home and basic services). These data were obtained from the database of the National Institute of Statistics and Geography (INEGI, acronym in Spanish, Instituto Nacional de Estadística y Geografia) [17]. 

Income Per Capita. Income per capita was determined from databases of basic indicators and economic information of the INEGI [17]. 

Mobility. We considered the variable of mobility as a proxy of the population’s adherence to infection containment measures that were implemented by the Mexican states. This variable was determined based on records of local mobility published by Google [18], which measure percent changes in the mobility of persons. These records show tendencies of daily movements, classified into distinct categories of places such as recreation centers, food stores, pharmacies, parks, public transport stations, workplaces and residential areas. We used weekly averages of estimated daily percent changes in mobility.

### 2.3. Descriptive Analysis 

SPSS IBM Statistics Version 25.0 (Armonk, NY, USA) was used for the present analyses. For the descriptive analysis, frequencies, percentages and measures of central tendency were used according to the nature of the variables. For cases of COVID-19, we used weekly measures of new and accumulated cases. The cumulative number of policies that were implemented by each state during the period of study for containing the epidemic was determined for each epidemiological week (from weeks 13 to 36). 

### 2.4. Panel Data Model

We applied a random effects panel data model in order to measure observable differences among states and control for non-observable variables that are constant across time [19]. The panel data model considers the evolution or change across time and assumes that there is heterogeneity with respect to an individual state’s inherent characteristics. Therefore, this model assumes that there is a difference of intercept for each individual state, and the intercept is a random variable. In the random effects model, there are two residual components. The first is the residual as a whole, where the residual is a combination of cross-section and time series. The second residual is an individual residual, which is a random characteristic of the *i*-th unit observation and remains at all times. We used a balanced panel that considered a tracking time from epidemiological weeks 13 to 36 for 31 Mexican states. That is, the analyzed sample had 24 observations for each state, obtaining a total of 744 observations.

The general model that we propose is given by:

(3) $$Y_{p,i}=\beta_{0}+\beta_{1}Policies_{p,i}+\;\beta_{2}GDP_{p}+\beta_{3}Population_{p}+\beta_{4}Density_{p}+\beta_{5}Health_{p}+\beta_{6}Poverty_{p}+\overset{j}{\underset{j=1}{\sum }}\beta_{j}Mobility_{j,p,i}$$

where $Y_{p,i}$ is the doubling time in state p for the epidemiological week i;

$Policies_{p,i}$ is the policy index in the state p in epidemiological week i;

$Mobility_{j,\;p,i}$ is the mean mobility j in epidemiological week i;

$GDP_{p},\;Population_{p},Density_{p},Health_{p}\;and\;Poverty_{p}$ are the socioeconomic variables of the state p.

## 3. Results

### 3.1. Descriptive Results

We obtained 1585 official diaries released by 31 of the 32 Mexican states (records from the state of Queretaro were not obtained until November 2020 and, therefore, were not included in the analysis) (Table 2). States with a larger size and population density issued a larger number of policies—for example, the states of Jalisco (195 policies), Mexico City (119 policies) and Nuevo León (104 policies).

Table 2 shows the main demographic and socioeconomic characteristics of each state, along with the total number of policies, number of COVID-19 cases per capita and average doubling time for epidemiological weeks 13 to 36.

### 3.2. Categories of Policies Implemented in Response to COVID-19 in Mexico

Officially documented policies throughout the country were classified into categories of closure and containment, economic measures and health measures (see Table 1).

**Figure 1:**
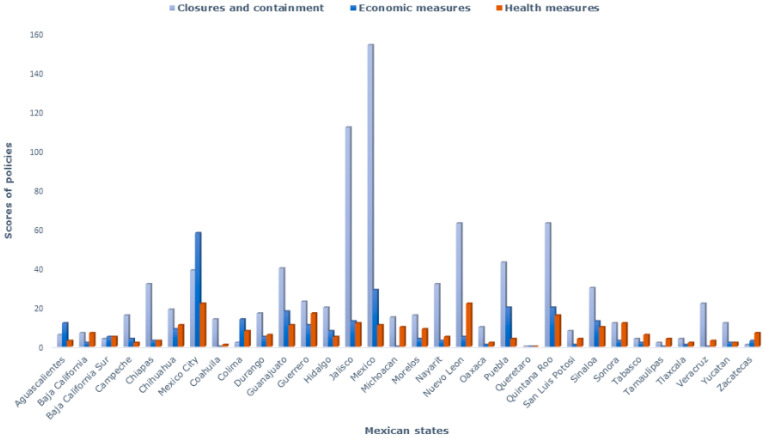
Categories of policies to face COVID-19 in Mexico.

Closures and containment (Policy categories C1–C8). Closing of work places (C2) was one of the principal documented measures in all of the Mexican states (41% of all policies), while other measures such as stay-at-home requirements (C6) or restrictions on gatherings (C4) represented a much smaller percentage of all policies. Notably, measures issued by state governments for reducing mobility (C7) represented 5.9% of all policies. The policy of school closures (C1) was emitted at the federal level and implemented nationwide; however, only three states mentioned this policy in their official publications (see Figure 1).

Economic measures (Policies E1–E3). A large proportion of states documented policies in this area, principally measures directed at income support (E2), but various policies having to do with debt assistance (E2) and fiscal measures (E3) were also implemented (Figure 1).

Health measures (Policies H1–H4). Compared to other types of policy, state governments documented relatively few specific health measures; these were largely related to public information campaigns (H1; 14%) and investment in hospital medical attention (H4; 12%)—see Figure 1.

The scores of all policies for each Mexican state, without rescaling, are shown in Figure 1.

**Figure 2:**
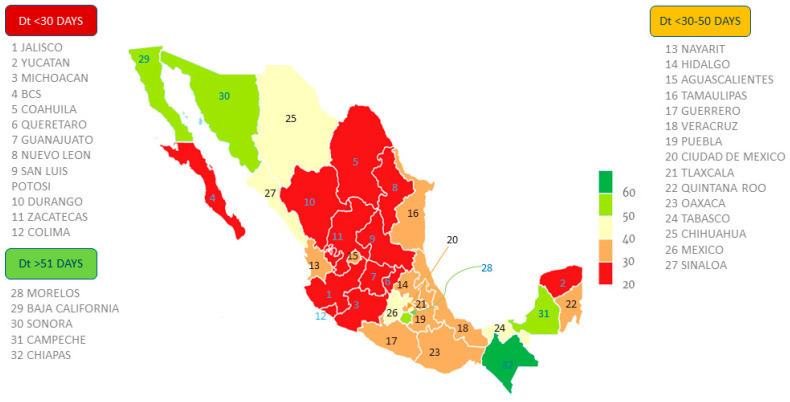
Mapping doubling time in Mexico. Note: Doubling time (Dt) average of the COVID-19 cases during epidemiological weeks 13–26 for each Mexican state.

To illustrate the average doubling time for each of the Mexican states across epidemiological weeks 13–36, we drew a map of Mexico. The strongest primary color (red) shows the Mexican states with the shortest doubling time, while the secondary green color indicates the Mexican states with the longest doubling time (Figure 2).

In general, states with a small population size (less than six million inhabitants) showed a slower COVID-19 transmission velocity. For example, the state of Campeche with a population of 899,931 had an average doubling time of 56 days, while the state of Jalisco, with a population of over 16 million, had a doubling time of 30 days. This general trend can also be observed in states with a lower policy index; nevertheless, Mexico City had an intermediate virus transmission velocity (41 days), which could be related to factors unique to this entity such as its extremely high population density compared to other states.

### 3.3. Relationship between Policy Index, New Cases and Doubling Time by Epidemiological Week for Each Mexican State

**Figure 3:**
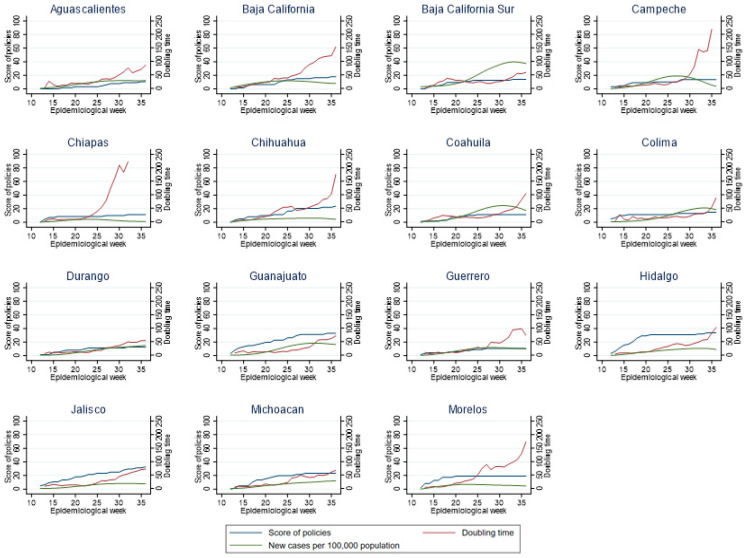
Policy index, new cases, and doubling time by week in states of Mexico (A).

**Figure 4:**
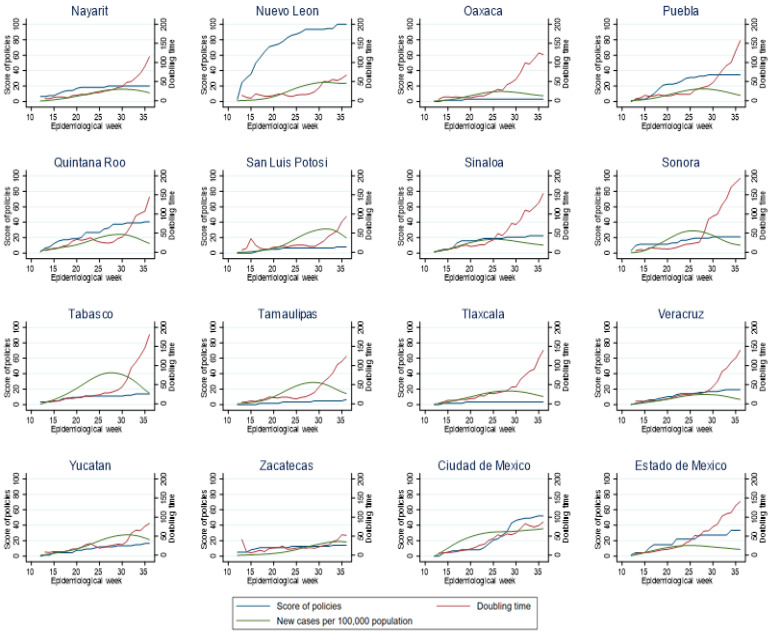
Policy index, new cases, and doubling time by week in states of Mexico (B).

Considering the dynamics of transmission velocity in individual states, we observed that those states that progressively issued policies, such as Mexico, Mexico City, Hidalgo, Morelos and Jalisco, were more successful in slowing the doubling time (see Figure 3 and Figure 4). In some states such as Durango and Colima (Figure 3), San Luis Potosi, Yucatan and Zacatecas (Figure 4), it can be observed that delayed implementation (or documentation) of policies was associated with peaks of viral transmission, while when policies were implemented, transmission velocity decreased.

In the states of Mexico, Chihuahua, Morelos, Puebla, Quintana Roo, Sinaloa, Sonora and Veracruz, it can be observed that the implementation of adequate policies directly impacted on the doubling time and the number of new cases (Figure 4). By contrast, in states such as Baja California Sur, Durango, Yucatan and Zacatecas, the number of policies that were implemented (as represented by the policy index) was very small, and a clear effect on the doubling time was not observed, Rather, the number of cases may have determined the growth of the pandemic curve and the doubling time. Moreover, in these states, the transmission velocity reached higher levels in less time (Figure 4). Mexico City showed that as the number of cases increased, so did the policy index; if indeed there was an effect on the doubling time, it was not very large (Figure 4) and was, perhaps, due to additional factors, as will be shown in the following analysis.

### 3.4. Panel Data Model

Two models were considered in the panel data regression analysis. Model 1 considered only the policy index and sociodemographic variables, while Model 2 considered these variables along with variables relevant to mobility.

*Model 1*. The panel data analysis revealed that prevalence of poverty and the health index strongly predicted a slower doubling time (regression coefficients of 2.405 and 1.417, respectively; Table 3, left column). Likewise, the policy index was significantly positively associated with lower velocity of viral transmission (doubling time; regression coefficient = 1.378; Table 3); in other words, an increase of one percent in the policy index score was associated with a 1.37-day increase in the doubling time. That is, greater prevalence of poverty, higher health index and greater policy index were all associated with an increase in doubling time (slower viral transmission) and a flattening of the epidemic curve. By contrast, population size and population density decreased the doubling time of the COVID-19 cases (Table 3).

*Model 2*. When mobility variables were included in the panel data analysis, poverty, the health index and the policy index remained significant positive predictors of doubling time, with regression coefficients of the health index and policy index decreasing in magnitude while that of poverty increased (Table 3, right column). In this model, income per capita emerged as a strong significant positive predictor of the doubling time of COVID-19 cases. Thus, our results indicate that an increase of USD 55 in income per capita increased the doubling time by 3.36 days. Population size was no longer a significant predictor, while population density remained a significant negative predictor of doubling time.

With respect to mobility variables, increasing mobility in parks by one percent was associated with a reduction in the doubling time of 1.10 days, and a one percent increase in mobility in public transport stations reduced the doubling time by 0.88 days. Strikingly, an increase in mobility within residential areas by one percent was associated with a decrease in doubling time of 4.19 days, making this the strongest negative predictor of doubling time. Thus, overall, increased mobility was significantly associated with more rapid viral transmission.

## 4. Discussion

The results of the present study show that the implementation of policies (represented by the policy index) was associated with slower propagation velocity (increased doubling time) of the COVID-19 pandemic. However, the panel data analysis clearly showed that other variables—the prevalence of poverty, income per capita, population density, health index and, especially, mobility—impacted the growth of the epidemic curve.

The aim of the present study was to assess the impact of the implementation of government policies on the doubling time of COVID-19 cases. We observed that all Mexican states established policies aimed at slowing the spread of COVID-19, and the principal measures were those centered on public information campaigns and closing of work places, in line with those measures established by the federal government. As expected, our data indicated that infection doubling time increased in association with the implementation of health policies. This relationship can be observed graphically in certain individual states and is further supported by the results of the panel data model analysis. However, the latter analysis revealed that the effect of policy implementation was modest compared to other factors that were examined. The relatively small effect of the policy index might have been due to several factors, including the influence of a number of cultural and sociodemographic factors, discussed below. One important consideration is that the individual states of Mexico are constitutionally autonomous with respect to the implementation of health policy [20], and we found that there was considerable variability in the states’ responses in this regard. Due to spread of the contagion through interstate travel, the lack of adequate policies in one state would negatively impact the efficacy of policies that may have otherwise been successful in bordering states.

Government policies can only be effective if they are followed by the population. There are a number of factors that could influence the population’s cooperation with governmental information campaigns, recommendations and mandates. Among these are education, trust in the government [21], the public’s exposure to erroneous information and the extent to which policies are enforced. In the present case of Mexico, close communication has generally been maintained between the federal government and the population during the pandemic, and a large percentage of policies have been focused on public in-formation campaigns. With respect to the enforcement of policy, optimal compliance to social distancing policies may require enforcement, and even then, compliance tends to decrease with time, which necessitates implementing additional measures [22,23,24]. In Latin American countries, an analysis showed that policies of obligatory confinement and use of masks in Colombia had a positive impact in controlling the epidemic, whereas in other countries such as Ecuador and Peru, these measures were not obligatory and enforced, and a positive impact was not observed [25]. In the case of Mexico, in general, public cooperation with containment and distancing policies was not obligatory or strictly enforced. Nevertheless, obligatory health control measures are not necessarily perceived as negative if they are preceded by a strong information campaign, such as that which was applied by the Mexican government, as well as confidence in the local government, which generates positive behavior and increases confidence in authorities [26]. Aside from these factors, a number of socioeconomic factors might make it impractical or impossible for some individuals to follow certain containment and closure policies.

Our analysis revealed important effects of the prevalence of poverty and the income per capita on the doubling time of COVID-19 cases. When mobility variables were considered in the panel data model, both of these factors were positively associated with the doubling time. The association of prevalence of poverty (percentage of population below the poverty line) with slower spread of infection could be related to a number of characteristics of this population. This sector of the population tends to be younger, which lowers the vulnerability to severe illness from COVID-19 [27], as well as generally being associated with rural areas of low population density. With regards to the policies that we documented, it was shown that the majority of states implemented measures of income support directed at this population with limited resources, which could partially explain a lower transmission velocity, although the effects of economic and social conditions on viral transmission velocity have not been established with certainty [28]. On the other hand, per capita income was also independently associated with slower infection rates. This seemingly contradictory result might be explained by considering that individuals earning a higher income tend to have more possibilities for working from the home, while those with lower incomes are more likely to have employment that requires them to work outside of the home and to use public transport [7]. Thus, for those with a lower income, it may not be possible to adhere to stay-at-home and social distancing policies. In Latin American countries, a clear influence of poverty and urban populations on the COVID-19 death rate among confirmed cases has not been observed [25]. However, in other countries, the epidemic tends to maintain a greater growth rate in urban populations with lower rates of poverty.

The health index (based on average life expectancy) was also found to be an important positive predictor of doubling time. It is important to point out that, since 2018, the Mexican government has initiated policies, such as universal healthcare coverage, that support disadvantaged sectors of the population and are in line with the philosophy that assertive response measures should be based on principles of equity and solidarity [8,29]. Such policies might indirectly provide some protection from future pandemics, potentially slowing the spread of infection as a result of improving the general health of the population.

Population size and mobility were two of the most important positive predictors of doubling time, both being associated with faster spread of infection. With respect to these characteristics, the Mexican states vary widely: Mexico City is a federal entity with the largest population size and density, with 5966 inhabitants/km^2^, while the state of Baja California Sur has the lowest population density of only 10 inhabitants/km^2^. The population of Mexico City is also extremely mobile: 22 million people are mobilized daily, which is equivalent to one-sixth of the entire population of the country. Moreover, public transportation in Mexico City is used extensively. Due, at least in part, to the extreme population density and mobility, it was necessary to implement a greater number of policies in Mexico City, including containment measures and, above all, investment in hospital attention and follow-up of cases within the community. Such policies apparently improved response capacity [30,31], which was, in turn, reflected in a slower transmission velocity and a greater capacity for medical attention in hospitals, which have not reported occupation rates of more than 75% of total capacity.

The present study has some important limitations. First, the doubling times were calculated based on published case number data. Considering the low per capita rate of testing that has been carried out in Mexico, these data on case numbers are most certainly an underestimate of the actual number of infections. Second, we did not attempt to distinguish between policies in order to determine whether individual policies may have differed with respect to their association with changes in doubling time, nor did we attempt to make between-state statistical comparisons. Third, we have no data on the extent to which the public followed the implemented policies. 

## 5. Conclusions

The present results underscore the importance of considering key sociodemographic variables such as income per capita, health index, population size and mobility when formulating public health policies in response to the current and future pandemics. The present results indicate that certain characteristics such as high mobility confer a greater risk to the rapid spread of disease [32]. In order to address this problem locally, targeted restrictions on mobility could be implemented, such as limiting access to public parks; at the state and national levels, mobility between regions of high and lower population densities could be discouraged or restricted. Areas such as public transportation hubs could be venues for public information campaigns and vaccination programs, thereby reaching the particularly vulnerable mobile population. The observed association between health index and slower doubling time suggests that a greater investment in general healthcare according to the necessities of the target population will be required in order to improve the responsiveness to future pandemics. Overall, policies should adopt a systemic, holistic and preventative focus in order to make an efficient system for confronting health problems.

In spite of the measures that have been implemented, the pandemic continues and, indeed, is showing a “second wave” of exponential increases in case numbers. In response, some states are implementing stricter policies that are, in some cases, punitively enforced. Policies of case detection and monitoring have been implemented at a large scale, which should allow for a better control over the epidemic [31], and a systematic vaccination program has begun in Mexico. It will be important to carry out further analyses in order to determine how these additional measures, especially the vaccination program, impact the COVID-19 pandemic.

## Figures and Tables

**Table 1:** Categories, values and total number of policies to contain the health emergency due to COVID-19 in Mexico.

Policy Categories	Policy Value ^a^	Total Policies ^b^
Closures and containment (C)		
School closing (C1)	7.56	5
Workplace closing (C2)	5.89	664
Cancel public events (C3)	8.89	34
Restrictions on gatherings (C4)	8.44	36
Close public transport (C5)	4.67	1
Stay-at-home requirements (C6)	7.33	1
Restrictions on internal movement (C7)	7.11	96
Restrictions on international movement (C8)	8.78	5
Economic measures (E)		
Income support (E1)	5.33	140
Debt/contract relief for households (E2)	5.22	12
Fiscal measures (E3)	4.43	117
Health measures (H)		
Public information campaigns (H1)	9.56	223
Testing policy (H2)	8.44	10
Contact tracing (H3)	9.0	9
Emergency investment in healthcare (H4)	9.78	61

^a^ The policy values range from 0 to 10, where 10 is the most relevant value to contain the transmission of COVID-19; ^b^ total policies correspond to the absolute number of documented policies.

**Table 2:** Sociodemographic characteristics and doubling time by Mexican state.

Mexican State	Population Size	Population Density	Health Index	Income per Capita	Doubling Time Average	New Cases per 100 k Population	Total Cases per 100 k Population
Aguascalientes	1,312,544	234	0.856	2737	34	19	486
Baja California	3,315,766	46	0.858	2730	55	22	552
Baja California Sur	712,029	10	0.857	3172	28	49	1220
Campeche	899,931	16	0.839	2200	56	26	644
Chiapas	5,217,908	71	0.83	1223	118	5	122
Chihuahua	3,556,574	14	0.849	2492	46	10	259
Mexico City	8,918,653	5966	0.865	3648	41	50	1256
Coahuila	2,954,915	19	0.853	2580	29	32	811
Colima	711,235	126	0.849	2434	22	23	584
Durango	1,754,754	14	0.844	2013	24	17	431
Mexico	5,853,677	191	0.844	2128	50	18	460
Guanajuato	3,533,251	56	0.813	1353	26	25	622
Guerrero	2,858,359	137	0.842	1789	35	19	472
Hidalgo	7,844,830	100	0.849	2792	32	16	400
Jalisco	16,187,608	724	0.848	2215	30	12	292
Michoacán	4,584,471	78	0.838	1967	29	15	384
Morelos	1,903,811	390	0.844	1982	55	12	290
Nayarit	1,181,050	42	0.847	2221	31	18	458
Nuevo Leon	5,119,504	80	0.857	3181	26	26	656
Oaxaca	3,967,889	42	0.827	1457	41	15	374
Puebla	6,168,883	180	0.837	1798	41	19	472
Queretaro	2,038,372	174	0.852	2829	42	29	375
Quintana Roo	1,501,562	34	0.851	2616	42	29	733
San Luis Potosi	2,717,820	44	0.839	2145	26	31	767
Sinaloa	2,966,321	52	0.844	2559	50	23	584
Sonora	2,850,330	16	0.849	2762	56	33	815
Tabasco	2,395,272	97	0.843	1820	45	50	1250
Tamaulipas	3,441,698	43	0.846	2267	35	31	784
Tlaxcala	1,272,847	318	0.845	1859	39	22	548
Veracruz	8,112,505	113	0.834	1497	39	15	381
Yucatan	2,097,175	53	0.837	2301	30	31	774
Zacatecas	1,579,209	21	0.842	1751	23	16	393

1 USD = 18.21 MXN (Mexican peso); Population size = Number of inhabitants; Population density = Number of inhabitants/km^2^.

**Table 3:** Panel data model: Effect of variables of interest on doubling time in Mexican states.

Variables	Doubling Time (Model 1)	Doubling Time (Model 2)
Policy index	1.378 ***	0.601 ***
	(0.163)	(0.168)
Population density	−0.00708	−0.0120 **
	(0.00523)	(0.00528)
Population size (log)	−1.922 *	−1.212
	(1.068)	(1.094)
Health index	1,417 **	1134 **
	(693.4)	(700.6)
Population poverty (%)	2.405 ***	3.290 ***
	(0.794)	(0.804)
Income per capita (in thousands)	1.006	3.366 ***
	(0.958)	(0.978)
Mobility in parks		−1.105 ***
		(0.389)
Mobility in transit stations		−0.883 **
		(0.376)
Mobility in workplaces		0.821 **
		(0.403)
Mobility in residential areas		−4.195 ***
		(1.460)
Constant	−1.362 **	−1.168 *
	(611.1)	(616.2)
Observations	739	739
Number of states	31	31

Notes: Standard errors in parentheses; *** *p* < 0.01, ** *p* < 0.05, * *p* < 0.1; Mobility: Patterns of movement over time by geography, in different categories of places. Regression coefficients are shown, corresponding to models not considering (left column) or considering (right column) mobility.

## Data Availability

Data sharing is not applicable to this article.

## Supplementary Materials

Supplementary File S1: Policies in states of Mexico, Supplementary File S2: Value for each policy by panel of experts, Supplementary Files are available online at https://drive.google.com/drive/folders/1rfkqFP6157ibLv5_P1auBT-rrZGV2WsB?usp=sharing.
